# Behavioral and Physiological Differences between Working Horses and Chilean Rodeo Horses in a Handling Test

**DOI:** 10.3390/ani9070397

**Published:** 2019-06-29

**Authors:** Paula Rosselot, Tiago Mendonça, Igor González, Tamara Tadich

**Affiliations:** 1 Departamento de Fomento de la Producción Animal, Facultad de Ciencias Veterinarias y Pecuarias, Universidad de Chile, Santa Rosa 11735, La Pintana, Santiago, Chile; 2 Behavioral and Physiological Mechanisms of Adaptation Department, Research Institute in Semiochemistry and Applied Ethology (IRSEA), 84400 Apt, France; 3 Programa de Magíster en Ciencias Animales y Veterinarias, Facultad de Ciencias Veterinarias y Pecuarias, Universidad de Chile, Santa Rosa 11735, La Pintana, Santiago, Chile

**Keywords:** equine, welfare, heart rate variability, behavior, working horse, Chilean rodeo horse

## Abstract

**Simple Summary:**

Animal welfare is a current societal concern, and non-invasive indicators are required to assess the welfare state of animals. The selection of horses for certain functions and individual differences could result in different strategies to deal with stressors. This is why in the present study we assessed behavioral and physiological responses of two types of horses (working horses and Chilean rodeo horses) to a handling test (bridge test). We evaluated five behaviors, the number of attempts, and the time required to cross a bridge. Heart rate and the variability of heart rate were registered with a polar system during rest and during the bridge test. Chilean rodeo horses displayed several active behaviors in order to avoid the bridge and required a higher number of attempts to complete the task, but physiologically responded better. On the other hand, all working horses crossed the bridge on the first attempt, without refusal behaviors, but physiologically did not respond as well as Chilean rodeo horses. Behavior does not always correlate with physiological data, and needs to be interpreted carefully when assessing horse welfare.

**Abstract:**

Non-invasive measures are preferred when assessing animal welfare. Differences in behavioral and physiological responses toward a stressor could be the result of the selection of horses for specific uses. Behavioral and physiological responses of working and Chilean rodeo horses subjected to a handling test were assessed. Five behaviors, number of attempts, and the time to cross a bridge were video recorded and analyzed with the Observer XT software. Heart rate (HR) and heart rate variability (HRV), to assess the physiological response to the novel stimulus, were registered with a Polar Equine V800 heart rate monitor system during rest and the bridge test. Heart rate variability data were obtained with the Kubios software. Differences between working and Chilean rodeo horses were assessed, and within-group differences between rest and the test were also analyzed. Chilean rodeo horses presented more proactive behaviors and required significantly more attempts to cross the bridge than working horses. Physiologically, Chilean rodeo horses presented lower variability of the heart rate than working horses.

## 1. Introduction

Animal welfare is a current societal concern, accompanied with an increase in awareness on how animals are raised and used. This creates a challenge for scientists, who need to generate information that can be useful to answer these concerns, but also contribute to the development of public policies based on science. For the assessment of animal welfare, this has resulted in the development of different evaluation protocols, based mainly on animal-based measures and also on individual differences that may arise as a result of different coping styles.

The term coping has become more common in the studies of the influence of environmental stressors on behavior and welfare [1,2]. Animals cope with environmental stressors through different mechanisms, among which behavioral and physiological adjustments, such as changes in heart rate variability (HRV), have been described [3,4]. Coping has been defined as the behavioral and physiological adjustments of an animal to master a situation [1,5]. A coping style is a coherent set of behavioral and physiological responses which are consistent over time and characteristic for a certain group of individuals [5]. Horses can then show specific coping strategies, revealing specific behavioral and physiological patterns to adapt to novel stressors [6].

On the basis of social stress research, two stress response patterns or coping styles have been suggested by Henry and Stephens [7]. The first type, known as the proactive (active) response, corresponds to the fight or flight response. Behaviorally, this response is characterized by territorial control, aggression, high resistance to restraint, and these individuals are more prone to take risks and form routines [1,5,8,9]. Proactive individuals respond physiologically with a strong sympathetic activation and an increase in noradrenergic stimulation [10]. The second type is known as the reactive (conservation-withdrawal) response characterized by behavioral immobility [11], in which the animal adopts a passive response, showing low levels of aggression if any [5]. These individuals respond to challenge with a strong hypothalamic-pituitary-adrenocortical (HPA-axis) and parasympathetic reactivity, increasing circulating glucocorticoids [10], and a higher baseline of non-enzymatic antioxidant capacity [12]. 

Since different coping styles involve particular responses of the autonomic nervous system (ANS), the use of variables associated with the variability of the heart rate (HRV) can be useful indicators [13]. From the HRV, the time domain analysis variables such as the mean beat-to-beat interval (mean RR), standard deviations of the RR intervals (SDNN), and root mean square of successive RR differences (RMSSD) have been proven to be a remarkable non-invasive measure of the ANS that can be applied in behavioral research to assess temperament and emotional states [13]. Moreover, frequency domain analysis can better discriminate between the contributions of the sympathetic nervous system (SNS) and the parasympathetic nervous system (PNS). This analysis includes two non-overlapping frequency bands [14]. The low frequency band (LF) of the HRV is influenced by the SNS, whereas the PNS is predominantly reflected in the high frequency band (HF). Considering this, it has been proposed that the low-to-high frequency ratio (LF/HF) is an index of the sympathovagal balance [15]. Moreover, the HF has been proven to be low during mental or physical stressful events [14].

Domestication has led to significant changes in the life of horses [16]. Housing and management can result in stress responses [17,18] when they do not meet horses’ needs. This in turn can induce changes in production efficiency, product quality, and performance. The intensity of these changes varies according to the specific husbandry system in which equines are kept, usually associated with their function or activity (sports, draught work, pleasure). Horses used for different functions could also have different welfare problems and coping strategies. When owners or caretakers select horses according to certain behavioral traits or according to their function, they could also be favoring certain coping styles.

In Chile, it is still common to see urban working horses in the peri-urban areas of cities where they perform draught work, transporting different types of products (wood, sand, vegetables) [19]. These horses are usually mixed breeds with an average height to the withers of 143 cm [19]. On the other hand, Chilean rodeo is a national sport, where Chilean creole horses are used (average height to the withers of 142 cm). The main physical effort of this sport consists of herding a steer within a circular arena (medialuna), and then pressing the steer against a padded wall with the horse’s pectoral muscles [20]. Chilean rodeo owners prefer certain behavioral characteristics that are usually associated with a more proactive coping style [21]. These horses are subjected to routines [22], and owners prefer horses that respond more actively to commands and when working with a steer, whereas owners of draught horses prefer horses that react minimally and remain calm when confronted with a stressor (i.e., traffic) [21] and are subjected to a more flexible environment [19], characteristic of a reactive coping style. 

In prior work, differences in basal levels of cortisol, oxidative stress indicators, and leukocytes, all parameters associated with stress response, were found between Chilean rodeo horses and Chilean urban working horses [21]. However, that study did not evaluate the differences between these two groups of horses when confronted with a potential stressor. The aim of this study was to investigate behavioral and physiological differences between Chilean rodeo horses and Chilean urban working horses when confronted with a handling test (bridge test).

## 2. Materials and Methods

### 2.1. Animals

A total of 26 horses, 13 working horses and 13 Chilean rodeo horses, were involved in this study. All horses were physically healthy, mares and geldings all over 5 years of age that had been actively performing either urban draught work (W-H) or Chilean rodeo exercise (R-H). All working horses were evaluated at the Veterinary and Animal Sciences Faculty of the University of Chile. All working horses arrived by trailer, and rested for at least 2 h before the start of the tests which were held between 10 a.m. and 4 p.m. All rodeo horses were assessed at the breeder’s farm during the same hours. To test these horses, the same bridge was constructed on the breeder’s farm. All procedures were approved by the institutional bioethical committee (certificate No. 12-2016).

### 2.2. Bridge Test (Handling Test)

The bridge test was designed as in Wolff et al. [23]. A bridge that was 1.5 m wide and 2 m long with a wooden floor and green iron rails at each side of 1 m height was used, and a starting line was set at a distance of 2 m before the bridge. The observer stood at a distance of 5 m (on the side) from where each horse was video recorded. An operator (the same for all horses) led the horse to the bridge and tried to make the horse cross it by pulling slightly on the rope if necessary. All horses were restrained and led with only a lead rope attached to a halter.

When the horse avoided walking on the bridge by going sideways or backwards, they were led back to the starting point and a new trial began. A trial was successful when the horse crossed the bridge with all four feet. 

### 2.3. Behavioral Measures

All horses were video recorded during the bridge test. The behavioral events observed were retreat (backward movement with at least one foot), swerve (sideways movement), jump (over the bridge), snort (forceful quick exhalation), and neigh (loud, prolonged high pitch call). Videos were then analyzed using The Observer XT (2011) software (Noldus Information Technology, Wageningen, The Netherlands). Offline continuous recording was used, and all behavioral event occurrences, number of trials, and the total time to cross the bridge were registered.

### 2.4. Physiological Measures

Heart rate (HR) and heart rate variability (HRV) recordings were obtained using a Polar Equine V800 heart rate monitor system (Polar Electro Oy, Kempele, Finland). For each horse, a 5 min long data set during resting time was recorded [13]. For this, after placing the polar system, a habituation to the monitor for 30 min was performed Then, a 5 min data set was obtained and the horse was led to the area where the bridge test was performed. A second data set from the beginning of the bridge test until its end was obtained. Additionally, heart rate was also recorded at the start of the bridge test (T1), when the horse crossed the bridge (T2), and 10 min after the bridge test (T3).

For HRV analysis, information from the Polar system was synchronized with the Kubios HRV software (Kubios standard 3.2.0, Biomedical Signal Analysis Group, Department of Applied Physics, University of Kuopio, Finland). Data were detrended using a smoothness parameter of 500 ms and artifacts were corrected by setting the custom filter to 0.3, according to Ille et al. [24]. Frequency band thresholds were established within each frequency interval using the following parameters: LF power = 0.01–0.07 Hz; HF power = 0.07–0.6 Hz [25,26,27]. The type of data extracted from each recording is shown in Table 1.

### 2.5. Statistical Analysis

Descriptive statistics (mean and standard deviation) were calculated for both behavioral and physiological measures. For physiological measures, normal distribution was assessed using the Shapiro–Wilk test. Subsequently, differences between sampling periods (rest and bridge test) within groups were calculated using the paired *t*-test or the Wilcoxon signed-rank test accordingly. For differences between groups (R-H and W-H) for each sampling period, the two-sample *t*-test and Wilcoxon rank-sum test were used. A *p*-value of <0.05 was established for significant differences. 

## 3. Results and Discussion

The bridge test is considered a restraint and human fear test. This test involves a combination of two potential stressors, namely, the procedure of handling and the fear towards humans [28]. Therefore, the demeanor and skill of the handler may make a significant difference, and the use of a familiar handler to the horse (owner, rider, or trainer) may have had a greater success in getting the horse to cross the bridge faster. In the present study, this method was used with the aim of comparing the behavioral and physiological responses between a group of working horses and a group of Chilean rodeo horses. The results suggest that the challenge of crossing an unknown bridge elicited different behavioral and physiological responses between the two groups.

### 3.1. Behavioral Measures

All working horses crossed the bridge on the first attempt, with a mean time of 9.77 ± 2 s. This group of horses did not display any of the assessed behaviors, with the exception of one horse that neighed before crossing the bridge. On the contrary, the Chilean rodeo horses required a significantly higher (*p* = 0.007) number of attempts to cross the bridge (1.77), varying from 1 to 4 attempts and an average time to cross of 19.81 ± 16 s (*p* = 0.09). Six rodeo horses required two or more attempts to cross the bridge, and these horses showed swerving and retreating when approaching the bridge and jumping once they started crossing it. Vocalizations such as snorts and neighs were present in three of these horses (Table 2). The time to complete the test was lower than the time reported by Wolff et al. [23], who reported times between 40 s and 10 min, although the distance from the bridge and the length of the bridge used in the present study were the same. Most studies have applied this test to compare the emotionality of horses of different ages, sex, or training status [4,23,29] and not according to function as in the present study. The behavioral results suggested that Chilean rodeo horses displayed more active (proactive) behaviors attempting to avoid the bridge by removing themselves from the stressor [30]. On the other hand, working horses passively accepted the handling and crossed the bridge (reactive) [2,31].

### 3.2. Physiological Measures

**Figure 1:**
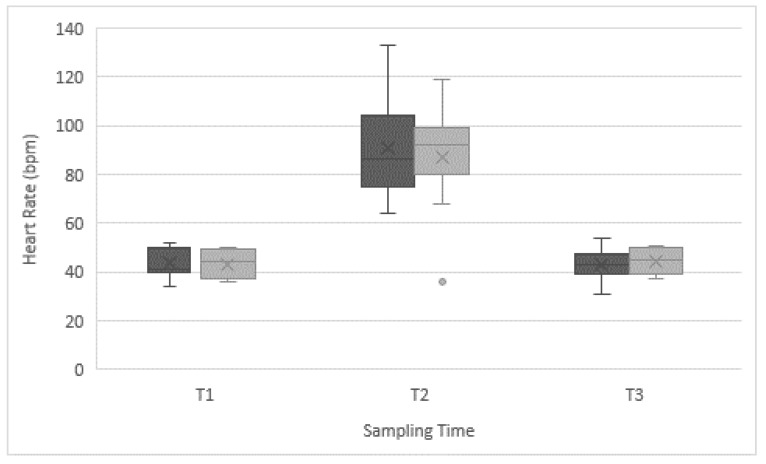
Box and whisker plot of the heart rate (HR) (bpm) of Chilean rodeo horses (dark grey) and working horses (light grey) at the start of the bridge test (T1), after crossing the bridge (T2), and 10 min after the bridge test (T3). Mean values, upper and lower quartiles, extreme values, and outliers are indicated.

A significant increase in HR between T1 and T2 was found in both groups of horses (*p* < 0.001 in both groups), recovering initial HR by T3 (10 min after crossing the bridge), but no differences between groups were found within any sampling time (Figure 1). The mean HR during the bridge test had a small, but significant rise in both groups of horses, but no differences were found between groups, neither for the minimum and maximum HR (Table 3). Fast alterations of the mean heart rate can occur within 5 s as a response to fear or excitement in horses and are attributed usually with short-term effects [4,13,32]. A rise in HR can be caused by an increase of the sympathetic activity, a decrease in vagal regulation, or changes in both regulatory systems [13]. Thus, the use of HRV can provide a better understanding of how the individual is coping with a stressor, since psychological states may have an impact on the sympathovagal balance in the absence of changes in HR [33].

The mean RR had a significant decrease in both groups when comparing the resting period with the bridge test period, as expected, with no differences between groups (Table 3). Contrary to Visser et al.’s [4] findings, our results showed an increase of SDNN between rest and the bridge test in the working horses’ group, and a significant difference between groups during the bridge test (Table 3). Thus, the overall variability of the HR during the bridge test was higher in the Chilean rodeo horses’ group than in the working horses’ group. Additionally, the working horses’ overall variability of the HR (SDRR) was higher during the bridge test than at rest, whereas the Chilean rodeo horses were more balanced between rest and the bridge test (Table 3). On the other hand, RMSSD decreased during the bridge test in both groups, but was significantly higher during the bridge test in the Chilean rodeo horses indicating a higher activity of the parasympathetic nervous system (PNS) in this group. The PNS has been associated with adaptive responses to the environment [34] and could indicate a better coping capacity for the Chilean rodeo horses. Visser et al. [4] also propose that individuals with a higher PNS activity would be more explorative and adaptive to environmental demands; our behavioral results would be in line with this since they displayed an active response in the bridge test.

The LF/HF ratio obtained from the power spectrum analysis is considered as an index of the cardiac sympathovagal balance [26] and has been proven to be a useful indicator of the sympathetic activity during physical and psychological stresses [13,35]. The HF power is thought to reflect PNS activity, whereas the LF power component should reflect both the sympathetic nervous system (SNS) and PNS [27]. Our results suggest a higher SNS activity in the working horses’ group than in the Chilean rodeo horses’ group in the bridge test. These results are in line with the RMSSD findings, showing that Chilean rodeo horses had a higher activity of the PNS, hence a better balance in terms of the autonomic nervous system (ANS) than working horses during the bridge test. In a future study, it would be important to consider a recovery time period, after the bridge test, in order to fully understand the coping mechanism in both groups.

### 3.3. General Remarks

Working horses took less time and passed the bridge at the first attempt, without displaying any active (proactive) behaviors in order to avoid the task. Nevertheless, physiologically they showed signs of emotional unbalance and a lower capacity to cope with the test. These results are in accordance with Yarnell et al. [36] and Squibb et al. [30], where it is proposed that behavior may not accurately reflect the internal affective state of horses. This could be related to the fact that horses are prey species and may mask behavioral signs of stress [36]. As in Squibb et al. [30], it is possible that the working horses that crossed the bridge were experiencing higher levels of stress, but also a greater stimulus control [37] than the rodeo horses that displayed more refusal behaviors. This higher stimulus control could be related to the fact that the bridge test requires handling by a person and the use of a halter that could act as the specific stimulus inducing a desired response (in this case, crossing the bridge) [37].

The differences in the responses between the two groups of horses could also be the result of breed differences and management conditions. Lesimple et al. [18] reported that breed and housing conditions appear to be of major importance in determining horses’ personality and remarked about the importance of considering how management practices can impact a horse’s reactivity. Working horses are also confronted daily with different environmental conditions and they could be accustomed to this exposure to novelty and not express flight behaviors. If this was the case, it should also be reflected in the ANS response, as in Górecka et al. [38], where horses used to novelty showed a lower HR response.

The emotional state of working horses should also be evaluated under tests that do not require human handling, such as the open arena and a novel object test [28]. A second plausible scenario, that requires further study, is as follows: since working horses are frequently exposed to stressful situations, poor husbandry practices, a high incidence of painful conditions such as lameness, and poor training systems, they could lose active control of situations resulting in learned helplessness [39]. According to Squibb et al. [30], an animal experiencing learned helplessness “abandons its attempts to cope and develops a dullness related to a decline in motivation and emotional response”. Although this study does not give evidence of an animal presenting learned helplessness, in situations where it does occur, this state would be a welfare concern, since individuals under this state have lost control of their environment.

## 4. Conclusions

Chilean rodeo horses displayed more active behaviors in order to avoid the bridge task. These behaviors were adaptive and efficient as physiologically these horses showed a better coping capacity. Working horses, that are frequently exposed to traffic, may have learned not to behaviorally respond to stimuli such as the bridge, creating an increase in reactivity in terms of their physiology. Our results are in accordance with other studies that show that an accurate interpretation of behavioral signs requires corroboration by means of physiological measures. This is of particular importance to take into account in current welfare assessment protocols where behavioral indicators are preferred as a non-invasive measure. Heart rate variability is an interesting, non-invasive tool, that provides physiological data. Heart rate variability in association with behavioral indicators may improve the emotional assessment of equines. 

## Figures and Tables

**Table 1:** Description of the time domain and frequency domain measures used in the present study.

Variable	Unit	Description
Time Domain Variables		
HR	bpm	Heart rate
RR	ms	R wave to R wave interval or inter-beat interval
SDNN	ms	Standard deviations of the RR intervals
RMSSD	ms	Root mean square of successive RR differences
Frequency Domain Variables		
LF	Hz	Low frequency band
HF	Hz	High frequency band
LF/HF ratio	--	Low frequency to high frequency ratio

**Table 2:** Exact time required for the bridge test, number of attempts, and frequency of occurrence of each behavioral event registered for each horse. Mean and standard deviation (SD) of each variable are also indicated.

Horse	Time (Seconds)		No. Attempts		Snort		Jump		Swerve		Retreat		Neigh	
	R-H	W-H	R-H	W-H	R-H	W-H	R-H	W-H	R-H	W-H	R-H	W-H	R-H	W-H
1	8	9.01	1	1	0	0	0	0	0	0	0	0	0	0
2	20	11.89	3	1	0	0	1	0	2	0	0	0	0	0
3	26	9.69	2	1	0	0	1	0	1	0	0	0	1	0
4	21.8	6.06	2	1	0	0	1	0	2	0	1	0	0	0
5	11.01	8.06	1	1	0	0	0	0	0	0	0	0	0	0
6	8.4	7.24	1	1	0	0	0	0	0	0	0	0	0	0
7	8.02	9.5	1	1	0	0	0	0	0	0	0	0	0	0
8	7.22	9.11	1	1	0	0	0	0	0	0	0	0	0	0
9	40.56	9.48	3	1	3	0	0	0	2	0	0	0	0	0
10	9.37	12.39	1	1	0	0	0	0	0	0	0	0	0	0
11	8.75	11.81	1	1	0	0	0	0	0	0	0	0	0	0
12	27.82	12.15	2	1	3	0	1	0	0	0	1	0	1	0
13	60.54	10.36	4	1	0	0	0	0	4	0	0	0	0	1

R-W, rodeo horse; W-H, working horse.

**Table 3:** The mean, standard deviation (SD), and *p*-values for each variable are provided according to horse group and to sampling time (rest or bridge test).

Variable	Time	R-H	W-H	*p*-Value
**Min HR (bpm)**	rest	38.61 (4)	40.15 (5)	0.405
	bridge	43.23 * (10)	41.54 (5)	0.962
	*p*-value	0.045	0.097	
**Max HR (bpm)**	rest	52.31 (8)	55.62 (11)	0.286
	bridge	91.06 * (21)	87.07 * (20)	0.431
	*p*-value	*<0.001*	*<0.001*	
**Mean HR (bpm)**	rest	43.35 (5)	45.10 (6)	0.392
	bridge	57.05 * (15)	53.56 * (8)	1
	*p*-value	0.001	0.001	
**Mean RR (ms)**	rest	1402.58 (181)	1354.88 (189)	0.442
	bridge	1110.33 * (244)	1152.82 * (225)	1
	*p*-value	<0.001	0.00	
**SDNN (ms)**	rest	67.52 (28)	54.03 (15)	0.234
	bridge	83.11 ^a^ (31)	71.58 ^b^* (18)	0.047
	*p*-value	0.142	0.001	
**RMSSD (ms)**	rest	73.97 (26)	59.50 (21)	0.208
	bridge	66.23 ^a^ (30)	54.38 ^b^ (17)	0.039
	*p*-value	0.208	0.393	
**LF/HF ratio**	rest	0.59 (0.6)	1.09 (0.9)	0.263
	bridge	1.46 * (1.9)	2.36 * (2.2)	0.064
	*p*-value	0.050	0.025	

* Indicates significant differences (*p* < 0.05) within groups (R-H and W-H) between sampling periods (rest and bridge test) for each variable. Different letters (a, b) indicate significant differences (*p* < 0.05) between groups (R-H and W-H) within a same sampling period (rest or bridge test) for each variable. R-H, rodeo horse; W-H, working horse; HR, heart rate; RR, R wave to R wave interval; SDNN, standard deviations of the RR intervals; RMSSD, root mean square of successive RR differences; LF/HF ratio, low frequency band to high frequency band ratio.
